# Understanding Senior Adults’ Needs, Preferences, and Experiences of Commercial Exergames for Health: Usability Study

**DOI:** 10.2196/36154

**Published:** 2024-04-05

**Authors:** Yu-Han Wang

**Affiliations:** 1 Department of Multimedia Design National Taichung University of Science and Technology Taichung City Taiwan

**Keywords:** exergame, senior user experience, senior technology acceptance, game technology, psychological perception, serious games, exercise, aging, older adults, physical activity

## Abstract

**Background:**

Many senior adults are at risk of mental and physical disorders due to a lack of sufficient exercise. Therefore, adherent exercise should be urgently promoted to improve senior adults’ muscle strength, preventing falls and conditions caused by physical and cognitive decline. However, off-the-shelf exercise games, so-called exergames, are mainly targeted at the younger generation or children, while senior adults are neglected, when this age group strongly needs exercise. Exergames could serve as a health intervention for promoting exercise.

**Objective:**

This study aimed to investigate senior adults’ experience, perceptions, and acceptance of game technology to promote exercise in order to suggest game design guidelines.

**Methods:**

In this usability study, participants engaged in playing Nintendo Switch and Xbox Kinect games, after which semistructured interviews were conducted. Before the gameplay, the participants provided their background information, exercise habits, and use of technology products. Next, all participants completed a workshop including 3 activities (brief instructions on how to play the games: 20 minutes; playing the selected exergames: 80 minutes; semistructured interviews: 20 minutes) for 2 hours a day for 3 days each. The participants played the latest Nintendo Switch games (eg, Just Dance, Boxing, Ring Fit Adventure) and Xbox Kinect games (eg, Kinect Adventures!, Mini Games). Just Dance, Zumba, and Boxing were played in activity 1; Ring Fit Adventure and Mini Games in activity 2; and Kinect Adventures! in activity 3. Reflexive thematic analysis was applied to identify the relative themes generated from the interviews.

**Results:**

In total, 22 participants (mean age 70.4, SD 6.1 years) were enrolled in the workshop in May 2021. The results of the generated themes included incomprehension of game instructions, psychological perception of game technology, and game art preferences. The subthemes generated from game art preferences included favorite game genres, characters, and scenes.

**Conclusions:**

There is a significant need for customized game tutorials considering senior adults’ cognitive and physical aging. Furthermore, the adventure game genre is preferable to other games. Humanlike game characters are preferable, especially those with a fit and healthy body shape. Nature scenes are more enjoyable than indoor stages or rooms. Furthermore, the game intensity design and playing time should be carefully planned to meet the World Health Organization’s criteria for physical activity in older adults. Intelligent recommendation systems might be helpful to support older adults with various health conditions. The guidelines suggested in this study might be beneficial for game design, exercise training, and game technology adoption of exergames for older adults to improve health.

## Introduction

### Background

The rapid growth of the aging population has drastically increased the need for medical and health care services. The life expectancy is increasing; however, the average life in a state of good health, the so-called healthy life expectancy, is almost 8-10 years less than the life expectancy [1,2]. It is important to increase the healthy life expectancy for active aging and reduce the financial burden on health systems caused by incapacitation, bedridden patients, and chronic physical or mental health conditions. The World Health Organization (WHO) [3] has suggested that adults 65 years and older perform 150-300 minutes of moderate-intensity or 75-150 minutes of strenuous-intensity aerobic physical activity in a week in order to improve physical and functional health and lower the risk of noninfectious chronic illness and mental health conditions. However, it has been reported that in Taiwan, 33.8% of older adults in the age group of 65-74 years and about 36% of 55-64-year-old “graying” adults do not exercise at all [4]. Many “graying” adults in Taiwan are at risk of mental and physical disorders due to a lack of sufficient exercise. Therefore, providing preventive health care and promoting adherent exercise are urgent issues to be considered in order to improve senior adults’ muscle strength and physical fitness to prevent falls and conditions caused by physical and cognitive decline.

### Studies on Exergames for Health

Several studies have highlighted the benefits and positive impact of exercise games, so-called exergames, on overall health. The term “exergame” is most frequently used by health-related researchers to refer to video games that use strength training, balance, or flexibility [5]. Exergaming has gained public and commercial interest due to its combination of fun and fitness together and is particularly popular for obesity-related interventions [6]. Furthermore, it is regarded as the future of fitness to maintain regular activity as it is a healthy, appealing alternative to other physical activities, as seen especially during the COVID-19 quarantine [7]. In addition to increasing physical exercise, exergaming could also be beneficial for psychological, cognitive, or psychosocial issues [8]. Exergames have been gradually applied in psychological treatment or physical rehabilitation for senior adults. For example, some studies have adopted exergames in psychological treatment for cognition [9], dementia [10], and depression [11]. Exergames have also been adapted for physical treatment to improve physical skills or fitness [12,13], balance [14,15], fall prevention [16], and rehabilitation [17-19]. Although many studies have applied off-the-shelf exergames for mental and physical health, with proof of their benefits and effectiveness, older adults’ perceptions, experiences, and expectations are rarely discussed. Therefore, it is important to investigate older adults’ experiences and expectations of playing exergames as a preventive approach to promote exercise habits.

### Exergame Technology

Exergames require players to interact with the virtual gameplay by means of their physical movements and gestures in the real world [20]. Exergames generally use motion-sensing technology, which tracks and monitors players’ movements while they perform game tasks using sensory devices, such as Nintendo’s Joy-Con or Microsoft’s Xbox Kinect. Motion-sensing technology differs between Nintendo and Xbox consoles. The Joy-Con contains an accelerometer and a gyroscope built into the device to track a player’s motion. Additionally, the Joy-Con also contains 3D touch technology, which provides subtle vibration feedback to the player. In contrast, Xbox Kinect, which is a camera-based and infrared depth-scanning-approach device, enables a player to interact with the game with body movements. Various types of game consoles with different underlying body motion technologies offer different user experiences, but the amount of physical effort and energy required to play exergames remain similar [21]. Accessibility and entertainability are the 2 distinguishing factors of exergames in promoting exercise and activity [22]. This study explored which interactive technology is preferred by older adults.

### Technology Acceptance of Older Adults

For decades, many studies have researched how people adopt technology [23-26]. Davis [23] first developed the technology acceptance model to explain user behavior and the intention to use technology. Chen and Chan [26] further modified the technology acceptance model, calling their modified version the senior technology acceptance model, to learn about senior users’ adoption of technology. The term “older adults” generally refers to people whose chronological age is 60 years or older [27]. Researchers suggest that older adults’ attitude toward using and their intention to use technology are particularly affected by their self-efficacy, anxiety, health conditions, cognitive ability, social relationships, attitude toward life and satisfaction, physical functioning, and the support of technology use they receive around them. The senior technology acceptance model has been applied to understand older adults’ behavior with regard to technology in Hong Kong [26], Sweden [28], and Taiwan [29].

According to a statistic survey by the American Association of Retired Persons (AARP), in 2019, in the United States, 44% of older adults enjoyed playing video games compared to 38% in 2016 [30]. The growing population of older adults in the game market shows that their needs should be considered in game design. However, many older adults may not have had much experience playing video games during their childhood, as computer-based video games became popular around the 1980s [31]. The target users of these exergames are mostly younger adults or children. The aging population is neglected in the market, when this age group strongly needs exercise. Therefore, this study investigated older adults’ experience, perceptions, and acceptance of game technology to promote routine exercise.

## Methods

### Study Design and Recruitment

The workshop was conducted in a spacious classroom that was transformed to accommodate exergame testing. The classroom was large enough to ensure there were no obstacles to the participants performing their movements and gestures while playing the games. Four Sanlux 55-inch 4K Ultra HD televisions with Nintendo consoles and Xbox Kinect consoles were set up in the classroom, and each television equipped with the consoles was shared by 2 participants while playing the selected exergames. The participants had sufficient space to move around and had full range of motion with the whole body. The classroom accommodated 6-8 participants in each workshop, and 3 workshops were conducted in total.

The inclusion criteria for the workshop and interview were participants aged 60 years and older who could exercise and understand the instructions provided. A total of 22 senior adults (≥60 years old) were enrolled in this study.

### Gameplay

**Figure 1:**
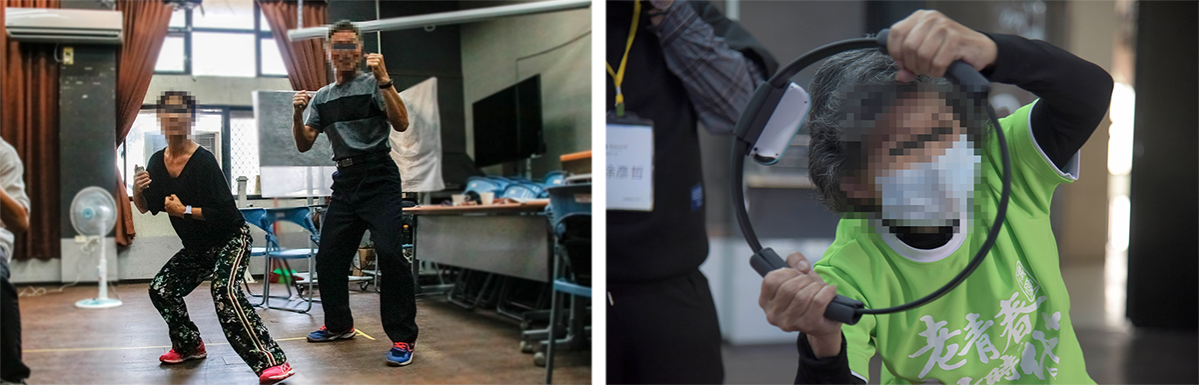
Participants played Boxing (left) and Ring Fit Adventure (right).

Each of the 3 workshops included 3 activities (Figure 1). Every activity was conducted for 2 hours a day for 3 days. Before playing the games, a questionnaire was administered, in which the participants provided their background information, exercise habits, and use of technology products. The following games were selected for this study: the latest Nintendo Switch games *Just Dance*, *Boxing*, *Mini-Games*, and *Ring Fit Adventure* and the Xbox Kinect game *Kinect* *Adventures!* Before each activity, the participants were provided with brief instructions for 20 minutes on how to play the games. Next, they played the selected exergames for 80 minutes, and finally, a 20-minute semistructured interview was conducted. The participants played *Just Dance* and *Boxing* in activity 1, *Ring Fit Adventure* and *Mini-Games* in activity 2, and *Kinect Adventures!* in activity 3.

### Data Analysis

Data were collected from the semistructured interviews using audio recordings and transcribed verbatim. Qualitative data were analyzed using thematic analysis, a qualitative data analytics method proposed by Braun and Clarke [32,33] and further clarified and defined as reflexive thematic analysis. Reflexive thematic analysis is a common approach to identify, analyze, and report themes from qualitative data sets in content analysis and grounded theory [34]. Braun and Clarke [35] have provided a 6-phase process of thematic analysis for researchers to conduct qualitative studies: (1) familiarizing with the data, (2) generating initial codes, (3) searching for themes, (4) reviewing potential themes, (5) defining and naming themes, and (6) producing a report. The 6-phase process is not linear but an iteratively developing process that is flexible enough to be tailored for qualitative data [36]:

- Phase 1 (familiarizing with the data): In phase 1, the researcher read and reread the data and made notes on a manual transcript. The researcher also identified connections between participants, data, and existing studies.
- Phase 2 (generating initial codes): The interview transcript was systematically analyzed through coding. Some codes matched the participants’ concepts, while others had to be interpreted by the researcher.
- Phase 3 (searching for themes): All the coded data were identified and clustered into broader topics or themes based on their similarities and overlaps.
- Phase 4 (reviewing potential themes): All the generated themes were reviewed to check whether they worked meaningfully and relevantly with the coded data.
- Phase 5 (defining and naming themes): After reviewing possible themes, the identified themes were subsequently defined and labeled.
- Phase 6 (producing a report): The generated themes answered the research questions about senior adults’ experience, perceptions, and acceptance of adopting exergames for exercise.

### Ethical Considerations

This study was approved by the Central Regional Research Ethics Committee China Medical University Taiwan (review number: CRREC-109-090). All participants were informed of the procedures involved in the study. They agreed to participate in the study and signed the consent form. The data in this study is anonymized. This study offered non-monetary incentives to participants as compensation.

## Results

### Participant Details

In total, 22 participants (n=16, 73% female; n=6, 27% male) were enrolled in the study. Their ages ranged from 60 to 82 years (Table 1).

**Table 1:** Participants’ general information (N=22).

Characteristics		Value
**Age (years), mean (SD)**		70.4 (6.1)
	Male	68.7 (5.4)
	Female	71.0 (6.4)
**Sex, n (%)**		
	Male	6 (27)
	Female	16 (73)
Education (years), mean (SD)		11.5 (4.5)
**BMI (kg/m^2^), mean (SD)**		
	Male	23.3 (4.5)
	Female	22.4 (2.8)
**Exercise routine, n (%)^a^**		
	Everyday	6 (27)
	3-5 days a week	12 (55)
	1-2 days a week	3 (14)
	1-3 days a month	1 (5)
**Exercise time (minutes/day), n (%)**		
	>30	16 (73)
	20-30	4 (18)
	10-20	1 (5)
	<10	1 (5)
**Exercise intensity, n (%)**		
	Barely there	11 (50)
	Moderate	10 (46)
	Harder	1 (5)
**Technology products use habits (possession of smartphones/tablets/computers), n (%)**		
	I do not have any of them.	1 (5)
	I have 1 of them.	8 (36)
	I feel comfortable using them.	13 (59)
**Frequency of using the technology products, n (%)**		
	Never	1 (5)
	1 time a week	3 (14)
	2-3 times a week	0 (0)
	Many times a week	1 (5)
	1 time a day	2 (9)
	Many times a day	15 (68)
Experience of playing videogames/digital games (arcade, home console, handheld game console, computer game, or online game), n (%)		12 (55)

^a^The percentages might add up to more than 100 because of rounding.

The background information of the 22 participants showed that only 7 (32%) met the WHO criteria for weekly exercise. The participants were familiar with phone technology and often used it for social media, photography, Google searching, or online shopping. Some of them had previous experience of playing video games. These results showed that the participants were less active and were comfortable using technology products.

Most participants performed regular or mild light-intensity exercise; however, only a few participants met the WHO criteria for senior adult exercise. WHO recommends multicomponent physical activity at a moderate or high intensity [3]; however, the results showed that the exercise intensity of older adults and the time they spend are not enough.

### Thematic Analysis

The semistructured interviews raised topics relating to user experience, psychological perceptions, and the adoption of game technology. In phase 2 of the thematic analysis, the manual transcript was systematically analyzed through coding. For example, “The tutorials in the game were not clear and not easy to understand. It is better to have instructors to teach me how to operate the game” was coded as the game tutorials not being supportive, being difficult to understand, and causing the participant to lose the motivation to play. In phase 3, the coded data was grouped into larger themes based on how they were connected. For example, coded data such as “do not know how to play,” “not understanding,” and “forgot tutorials” were clustered into “incomprehension of game instructions.” In phase 4, the identified themes were carefully reviewed to ensure they accurately and meaningfully represented the coded data. In phase 5, the themes were defined and named as (1) incomprehension of game instructions, (2) confusion caused by a complicated interface, (3) frustration caused by the fast game speed, (4) a sense of control and freedom, (5) psychological perception of game technology, (6) social interaction, and (7) preference of game art. These 7 themes could answer the research questions about older adults’ experience, perceptions, and acceptance of exergaming. The first 6 themes and the participants’ feedback are shown in Table 2.

**Table 2:** Themes 1-6 generated from the interviews.

Theme and example quotes		Sex (male/female), age (years), game experience (yes/no)
**1. Incomprehension of game instructions (n=8, 36%)**		
	“It is very critical that someone could guide us before playing games [*Just Dance*]. Knowing how to play motivates me to play the game.”	Female, 74, no
	“I need someone next to me and tell me how to play.	Female, 67, yes
	“I don’t know how to play the game because I don’t understand the game mechanics and rules.”	Male, 68, yes
	“I need a detailed game tutorial of the game to support me when playing the game.”	Female, 75, no
	“The tutorial in the game is not clear and not easy to understand. It is better to have instructors to teach me how to operate the game [*Ring Fit Adventure*].”	Male, 62, yes
	“By the game instructions, I cannot fully understand what it means and how to play. I prefer someone (instructors) to teach me for better understanding.”	Male, 72, yes
	“I can't remember things well. So, I forgot tutorials while playing. I might remember how to play after many times gameplay.”	Female, 79, yes
	“I need more practice to remember how to play.”	Male, 76, yes
**2. Confusion caused by a complicated interface (n=5, 23%)**		
	“At the beginning, it was difficult to understand the game interface, but after practicing, I got used to controlling it.”	Female, 66, no
	“I don’t know the meanings of the icons in the game interface.”	Female, 74, no; male, 76, yes
	“There is too much information on the screen; I don’t know what to look at while playing [*Ring Fit Adventure*].”	Female, 75, no
	“The game interface was very complicated and difficult to use. I guess it is also difficult to control machines for most of the older adults [*Just Dance*].”	Female, 63, no
**3. Frustration caused by the fast game speed (n=8, 36%)**		
	“I would give up and feel frustrated when I cannot keep up with the speed of the games.”	Female, 82, yes
	“I wish I could control the speed of the game from slow to fast so that I could participate in the game, not be excluded since the very beginning.”	Female, 63, no
	“I was too slow in the response in the playing. For example, I wanted to jump and get the golden coin, but when I jumped, the golden coin disappeared [*Kinect Adventures!*].”	Female, 78, yes; female, 75, no
	“It is very difficult and complicated to do more than 2 actions at the same time. I have to avoid getting bombed and to get golden coins [*Ring Fit Adventure*].”	Female, 74, no
	“I feel I am not as agile as I was. The game should provide different levels of difficulty for different ages.”	Female, 79, yes
	“After seeing the boxing icon showing up from the bottom of the screen, I need to punch at the right tempo. But I was always too late to react. I need some time to think and then do the action [*Boxing*].”	Female, 66, no
	“The speed in the game was too fast for me. I think the game should provide different levels of difficulty or provide different levels according to different age groups [*Boxing*].”	Female, 79, yes
**4. A sense of control and freedom (n=4, 18%)**		
	“I like to use joystick to control games because of the tactile responses, although I feel free when I don’t need to hold anything to control the game. However, I don’t receive tactile sensation feedback to know whether I did the correct movement or not [*Kinect Adventures!*].”	Female, 60, yes
	“I like to use my body motion to control the game because I don’t like to hold anything on hands. I don’t know how to use joystick, and I feel it is quite complicated. It is easy, free, and intuitive to control the game by my body.”	Female, 63, no
	“I prefer the realistic feeling of pressing and dragging the Ring-Con. It gives me real feedback and is easy to use.”	Male, 71, no
	“The vibration of the joystick gives me feedback of correct movement, and I like to receive the feedback [*Boxing*].”	Female, 74, no
**5. Psychological perception of game technology (n=9, 41%)**		
	“Playing the games could make me keep focus, train my brain and coordination.”	Female, 66, no
	“It is so much fun to do exercise by playing games. I didn’t expect myself to enjoy doing exercises [*Boxing*, *Kinect Adventures!*].”	Male, 68, yes
	“I prefer to play *Boxing* game because it is very challenging.”	Male, 62, yes
	“I feel it is fashionable to play state-of-the-art games, which could connect me and the younger generations.”	Female, 82, yes
	“I feel I am keeping up with trends because I can operate game technology device and share with my grandchildren.”	Female, 74, no
	“I didn’t know games could so charming and interesting. It is a fresh experience, and it is quite fashion and trendy to play games for me.”	Female, 79, yes
	“I would like to try new things, and I wish I could keep up with the times.”	Female, 74, no
	“I was afraid of new technology in general. I don’t know how to use them. But if I have a chance to approach it and someone could teach me, I will be happy to learn.”	Female, 60, yes
	I feel video games are not for us; it is for the youth.	Female, 82, yes
**6. Social interaction (n=5, 23%)**		
	“It has become a common interest between me and my grandchildren. We can play together, and we have common topics to talk about.”	Female, 75, no
	“Collaborating with other players is very fun, and it attracts me to continue using exergames.”	Female, 69, yes
	“I am feeling that I don’t want to lose against my friends. I will make every effort to win.”	Male, 68, yes
	“I felt lonely when I did exercise alone, so I like exergames, which allow me to do exercise with people.”	Female, 68, no
	“I enjoyed playing with friends who are the same age as me. It’s much more fun to play games or do exercise with friends than by myself.”	Male, 63, yes

Additionally, the subthemes of game art preference were generated from the “preference of game art” theme. The qualitative data related to game art were collated and coded into game genres, characters, and scenes (Table 3). In the interviews, participants talked about their favorite games from among *Just Dance*, *Zumba*, *Boxing*, *Kinect Adventures!*, *Mini Games*, and *Ring Fit Adventure*. Adventure games were constantly mentioned as participants’ favorite games (n=16, 73%, participants): 11 (69%) participants preferred *Kinect Adventures!*, and 5 (31%) participants liked *Ring Fit Adventure*. Only 3 (14%) participants liked the *Zumba* dance game and 3 (14%) liked *Boxing*. Thus, most participants were fond of the adventure game genre compared to the sports genre, such boxing or dance. Most participants (n=13, 59%) preferred true-to-scale 3D human models as game characters instead of cartoons. Some participants (n=8, 36%) preferred to see a real man on the screen to lead them in exercise, that is, the participants preferred the game characters to be real-scale humans or virtual models. Nearly all participants (n=21, 95%) mentioned that they enjoyed and immersed themselves in outdoor or natural scenes in the games, while only 1 (5%) participant preferred a static virtual stage.

**Table 3:** Subthemes generated from theme 7 (preference of game art; n=8, 36%, participants).

Subtheme and example quotes		Sex (male/female), age (years), game experience (yes/no)
**7.1. Favorite game genre**		
	“My favorite game is adventure games. It made me focus and immersed myself in the gameplay. The adventure game is very exciting, which could also train my brain and improve my coordination. I don’t usually have the experience in my daily life [*Kinect Adventures!*].”	Female, 74, no
	“The *Ring Fit Adventure* provides a more intense and challenging exercise for me. I love to take the challenge.”	Male, 76, yes
**7.2. Preference of game character**		
	“I like the game character is made by 3D humanlike model with a realistic ratio, such as the character in *Ring Fit Adventure*. The character looks fit, muscular, and powerful.”	Female, 75, no
	“I prefer a real man in the game, such as the coaches in *Zumba*. They are real people, who make it easier to understand and learn their dancing.”	Female, 69, yes
**7.3. Preference of game scene**		
	“I like the game with outdoor natural scenes. I feel like I was doing exercise outdoor rather than in an indoor room.”	Female, 69, yes
	“I was very excited and fully immersed in the game with a natural scene, such as *Xbox Adventure!* or *Ring Fit Adventure*.”	Male, 63, yes
	“The natural background made me feel comfortable, and I wanted to go forward to see what shows up next. It also keeps me focus on the gameplay [*Kinect Adventures!*].”	Female, 69, yes
	“I prefer static stages in the background because then I can focus on my game tasks [*Boxing*].”	Female, 60, yes

### Themes Generated From the Interviews

A total of 7 themes were generated from the semistructured interviews. Of these themes, 3 (43%; themes 1, 5, and 7) were initially identified in the study. The remaining 4 themes (57%; themes 2, 3, 4, and 6) aligned with prior research findings and are discussed in the *Comparison With Prior Work* section.

#### Theme 1: Incomprehension of Game Instructions

Lacking gaming experience in their youth might result in senior adults not understanding the game mechanics, the meaning of visual effects or reward icons, health points (HPs), or experience points (EPs). The instructions in the gameplay were not easy to understand, so most of the participants perceived them negatively. Moreover, although some participants had game experience, it did not seem to support their learning in exergames. They still needed appropriate instructions for gameplay learning.

As the game instructions were not helpful to the participants, they needed instructors to explain and teach them again to learn how to play. However, although the participants were taught how to play before the gameplay, they forgot how to play when playing. It was not easy for some participants to understand and memorize the given information. These findings showed that age-related visual and hearing loss might also contribute to increasing the barriers to understanding the gameplay. Thus, repetitive and real-time tutorials in games might be helpful to support memory, learning, and thinking. Therefore, simple visual instructions and oral guidance using plain language might support senior adults’ game learning.

#### Theme 2: Confusion Caused by a Complicated Interface

In general, younger adults tend to find game interfaces more intuitive and user friendly compared to senior adults with regard to playing digital games. Senior adults often encounter significant challenges when attempting to engage with game interfaces. For instance, *Ring Fit Adventure* integrates a diverse range of physical tasks within its exercise regimen. Players are required to perform actions such as pressing or dragging the ring with their hands while simultaneously engaging in activities such as jumping, squatting, or running with their feet. The information and tasks presented in the game interface pose a complexity that senior adults may find challenging to comprehend. For instance, 1 (5%) participant (female, age 75 years, nonexperienced player) said:

There is too much information on the screen, I do not know what to look at while playing.

Another participant (female, age 63 years, nonexperienced player) said:

The game interface is very complicated and difficult to use.

Furthermore, senior adults often struggle to grasp the meaning of icons or virtual objects displayed in the game interface. For instance, they may not be aware that collecting golden coins in *Ring Fit Adventure* can lead to earning additional points. Two participants expressed:

I do not know the meanings of the icons in the game interface.

#### Theme 3: Frustration Caused by the Fast Game Speed

One participant expressed frustration and a sense of exclusion when unable to keep up with the game’s pace. They desired the ability to control the game’s speed, allowing for a more inclusive gaming experience. The participant (female, age 66 years, nonexperienced player) playing *Boxing* noted that her ability to perform actions effectively was affected by slower response times. She explained:

After seeing the boxing icon showing up from the bottom of the screen, I need to punch at the right tempo. But I was always too late to react. I need some time to think and then do the action.

Another participant (female, age 82 years, experienced player) said:

I would give up and feel frustrated when I cannot keep up with the speed of the games.

In addition, participants perceived a decrease in agility compared to their previous exercise experiences, which could be attributed to the natural decline associated with aging. Thus, an inappropriate game speed could result in senior adults feeling frustrated and avoiding playing exergames.

The American College of Sports Medicine [37] has classified the characteristics of physical fitness into health-related and skill-related components. The skill-related components of physical fitness include agility, coordination, balance, power, reaction time, and speed, which can be adjusted in-game to meet individual conditions. The results in this study showed that game speed levels should be adjustable according to given skill-related health conditions. Senior adults need more time to understand what is going on and how to react accordingly. Therefore, games should provide a range of difficulty levels specifically designed to accommodate different age groups, thereby ensuring an enjoyable gaming experience for senior adult players.

#### Theme 4: A Sense of Control and Freedom

Participants shared diverse preferences regarding their choice of gaming controls. Some appreciated the tactile responses provided by a joystick (n=3, 14%) or the Ring-Con (n=7, 32%), while others found freedom in not having to hold anything when controlling the game, such as Kinect motion-sensing interaction (n=12, 55%). Alongside their appreciation for the sense of freedom, 7 (32%) participants favored the Ring-Con because it resembles holding a steering wheel, thus enhancing their perception of control.

However, the absence of tactile feedback in some games left participants uncertain about the accuracy of their movements. For example, 1 (5%) participant said:

Although I feel free when I do not need to hold anything to control the game...I do not receive tactile sensation feedback to know whether I did the correct movement or not.

#### Theme 5: Psychological Perception of Game Technology

Playing exergames was perceived to relate to enjoyment, socializing, achievement, frustration, defeat, and keeping up with trends. Generally, participants enjoyed playing and socializing with people. In addition, playing exergames and operating technological devices made them feel trendy.

I feel I am keeping up with trends because I can operate [a] game technology device and share with my grandchildren.

Female, 74 years

Some participants felt frustrated when they could not follow the game speed or obtained lower scores due to their longer reaction time and lack of coordination.

I want to have a feeling of achievement, rather than being defeated.

Therefore, the game design should consider senior adults’ psychological perceptions to meet their emotional needs.

#### Theme 6: Social Interaction

The use of exergames fostered a sense of connection and engagement, both within the family and among peers.

I enjoyed playing with friends.

Male, age 63 years, experienced player

I like to play with my family, and it is the biggest motivation to play.

Some participants enjoyed the collaboration and competition with team players. For instance, 1 (5%) participant (female, age 69 years, experienced player) expressed:

Collaborating with other players is fun, and it attracts me to continue using exergames.

These findings showed that playing exergames serves as a common interest that allows for enjoyable collaborative experiences, providing shared topics for discussion, and enhancing the motivation to continue using exergames.

#### Theme 7: Preference of Game Art

The game preference for game art and game genres was investigated. The exergames in the workshop included the latest Nintendo Switch games, such as *Just Dance* and *Ring Fit Adventure*, and Xbox Kinect games, such as *Kinect Adventures!* Among these games, 16 (73%) participants were fond of adventure games, including *Ring Fit Adventure* and *Kinect Adventures!* Interestingly, the results showed that younger senior adults (6/16, 37.5%, participants; average age 65.8, SD 5.7 years) preferred *Ring Fit Adventure*, which is a resistance exercise and a high-intensity training game, more than older senior adults (10/16, 62.5%, participants; average age 71.4, SD 5.3 years). Other participants (n=6, 27%) preferred less intensive exercise games, such as *Boxing* and *Zumba*. Of these 6 participants, 5 (83%) had no previous game experience, and the average age was 73.2 (SD 6.2) years. Therefore, younger senior adults might prefer a more intensive resistance exercise, while older senior adults might prefer a gentle exercise with less leg work.

For game characters, most of the participants preferred a human or humanlike character because it was easier to understand their movements. The participants also could reflect themselves as the avatar if the game character looked like a human. Of the 22 participants, 21 (96%) preferred human or humanlike characters, of which 13 (62%) participants preferred a humanlike game character and 8 (38%) preferred real humans as game characters. One participant did not show her preference. These findings showed that among the cute animal characters and human (humanlike) characters, senior adults prefer human or humanlike avatars, which allows them to easily reflect themselves in games and observe exercise movements more clearly.

For game scenes, outdoor nature scenes were the most preferable, such as scenes in *Kinect Adventures!* and *Ring Fit Adventure*. Of the 22 participants, 20 (91%) preferred nature scenes, followed by static scenes. The participants felt the nature scenes made them feel comfortable and helped them focus. Two participants preferred simple and clean colored indoor scenes rather than a sophisticated background, which allowed them to focus on the game mission and not be distracted by the background.

Therefore, adventure games, humanlike characters, and nature scenes could create a sense of reality and players might be more easily immersed in the gameplay.

## Discussion

### Principal Findings

In this study, most of the participants had no or little experience of playing exergames. However, after playing exergames in the workshop, they found them interesting and appealing. The results of this study reflect factors of the senior technology acceptance model [26]. Most of the participants referenced a positive attitude, perceived usefulness, and social relationships regarding using game technology. However, the participants provided negative feedback for the perceived ease of use and support of technology use. Lacking experience of playing video games might have also resulted in the participants not having the knowledge and skills needed to adopt game technology.

The supports for technology use, such as understandable tutorials or game mechanics, to help senior adult players are not sufficient. Thus, there is a significant need for customized instructions for senior adults. Overall, the reasons the participants did not have the intention to play could be because they thought video games are for younger people, not for them. Therefore, exergames should also meet senior adults’ psychological needs to increase their adoption of game technology.

The results of this study present older adults’ experience, perceptions, and acceptance of commercial exergames. In total, 7 themes were generated from the semistructured interviews; 4 of these are in line with previous studies, but 3 were identified in this study.

Three distinguished themes generated from this study are incomprehension of game instructions, psychological perception of game technology, and preferences of game art. These themes have rarely been discussed in prior studies. The psychological perception toward exergames that the participants expressed was that it is fashionable and trendy to play the latest digital games, which belong to the youth. They also enjoyed the gameplay by collaborating and competing with their peers. Among the exergames, the participants preferred adventure games much more than other genres. In this study, the adventure games were *Kinect Adventures!* and *Ring-Fit Adventure*, both of which focus on players’ action tasks. These types of games typically emphasize storytelling and character development, as players assume the role of a principal character who must overcome various challenges and obstacles to reach their objectives. The exciting experience of outdoor sports motivated them to continue to play, and the virtual environment provided a safe place to perform thrilling activities.

The game characters and scenes are also critical visual elements to immerse senior adult players in games. In this study, senior adults preferred humanlike characters who looked healthy, fit, and muscular. The game character with a healthy outlook inspired senior adult players to reflect themselves as energetic people exercising in the gameplay. The nature scenes in the game also gave the senior adult players feelings of comfort and relaxation during the gameplay. WHO recommends that adjustable intensity and game levels should be carefully designed to support senior adults perform a sufficient amount of physical activity.

However, if senior adults cannot understand how to play a game or react in time, they feel defeated and frustrated. They may give up playing straight away. Therefore, there is a significant need for game tutorials that are easy to understand for senior adults and game levels that are suitable for their mobile ability.

### Comparison With Prior Work

Four themes generated from the qualitative data demonstrated similar issues as in previous studies. First, “confusion caused by a complicated interface” (theme 2) and “frustration caused by the fast game speed” (theme 3) are in line with the results of Aarhus et al [38], who found that simultaneously increasing information, speed, and colors would more likely increase cognitive challenges. Although Aarhus et al [38] adopted Nintendo Wii in a physical rehabilitation context, the issue remains in the current Nintendo Switch. Second, the game speed was too fast to follow for most of the participants, which is in line with the results of Brox et al [39]. From the cognitive ability perspective, an age-related decrease in working memory causes a reduction in the amount and speed of information processing [35]. Third, the participants in this study highlighted “a sense of control and freedom” (theme 4) in the gameplay, similar to the results of Thin et al [21], who found that the game experience of motion-sensing games is preferrable due to its greater freedom and holistic movement experience. Fourth, “social interaction” (theme 6) is a prominent motivation for senior adults to adopt exergames, which is in line with previous studies [38]. Therefore, social interactions with peers, friends, the family, and society formed through playing exergames are appreciated.

Previous studies have also discussed the aforementioned themes, although most of the commercial games tested in previous studies were Nintendo Wii, Wii Fit, Wii Balance Board, and Wii Sports. Compared to prior studies, this study adopted the latest Nintendo Switch and Xbox Kinect to investigate the player experience. However, obstacles, such as game speed, interface, and tasks, remained, indicating that the latest games still do not consider the needs of senior adults.

### Evaluation of This Study

According to the criteria for scientific rigor (credibility, dependability, confirmability, and transferability), in the qualitative research proposed by Lincoln and Guba [40], consistent outcomes are expected when replicating the research process within the same setting. To enhance credibility in this study, continuous interaction was maintained with each participant throughout the data collection process. Furthermore, the participants were encouraged to provide examples while discussing their gaming behavior and experiences, with the interviewer posing follow-up questions. This approach facilitated the participants’ familiarity with both the research setting and the content, thereby ensuring an accurate interpretation of their original perspectives. Regarding transferability, which pertains to applicability, the researcher provided detailed descriptive data, such as participants’ demographics, exercise characteristics, technology use habits, inclusion criteria of recruitment, workshop and interview procedures, and the iterative research process. The information helped the researcher explain the participants’ behavior and experience within a gaming context, potentially making them meaningful and transferable to an external observer. To ensure dependability, which is related to consistency, a detailed analysis process was used throughout this study [41], thereby establishing the potential for reproducing the outcomes across similar participant cohorts and settings. Given the constraints, concerning confirmability, which relates to maintaining neutrality, although the absence of an external review was recognized, diligent steps were taken to compensate for this limitation by implementing a rigorous process of self-evaluation and critical reflection on the research process and outcomes. This included a careful examination of the data, consistent cross-referencing with established literature, and self-awareness regarding potential biases.

### Strengths and Limitations

This study makes a noteworthy contribution by emphasizing the importance of game tutorials, preferences of game art and genres, and the perception of trendiness in the game design for older adults. However, the study does have limitations. First, it is essential to acknowledge that the sample size was restricted to 22 participants, consequently impacting the precision of estimates for main outcomes. Second, evaluating the qualitative study’s confirmability would ideally involve external researchers. The advantage of having a single researcher code the content is that it ensures consistency and stability in the use of codes; however, confirmation bias can be manifest during various stages of the research process, such as data coding or interpretation [42]. As previously stated, a single researcher may hold preconceived ideas or preferences about the outcomes they anticipate or desire, and these biases can inadvertently impact their scholarly work. Although this study was conducted by a single author, the inherent limitations associated with solo authorship are recognized. The absence of external researchers in this study could limit the diversity of perspectives during data coding and interpretation. This limitation may result in a narrower scope of interpretation and analysis, potentially overlooking valuable insights. By acknowledging potential biases and preconceptions, steps have been taken to minimize their impact on the study’s findings. Although collaborative research may not have been feasible in this study, the research process involved continuous cross-referencing with established literature and a sustained awareness of potential biases. It is hoped that this study will contribute valuable insights to the field of digital exergames for senior adults, despite its single authorship limitations. Additionally, it is anticipated that future research will build upon the study’s findings to further enhance our understanding of senior adults’ experiences in engaging with exergames.

### Conclusion

Exergames could serve as an engaging approach to promote exercise and a healthy life among senior adults. Most of the prior studies have focused on usability and facilities, but senior adults’ psychological perception toward the exergame experience is highlighted in this study. The findings of this study have some important practical and research implications for adoption of game technology, as well as for research with senior adults’ gameplay experience for future work. First, tailored game tutorials for senior adults could be beneficial for increasing the adoption of exergames to promote physical health. Because of insufficient game experience in their youth and cognitive decline due to aging, there is a significant need for understandable and age-friendly tutorials of exergames to equip senior adults with affordable information and skills to get into the game scenario and mechanics. Second, preferences of game art and genres reveal that adventure games are the most favorable game genre and humanlike avatars in nature scenes are most liked among senior adults. Moreover, evidence from this study and the literature shows that the exercise time and intensity of senior adults in Taiwan are clearly not sufficient according to WHO criteria. Thus, game design should plan appropriate game times and intensities for senior adults, which could support them in gradually performing moderate- or high-intensity exercise to promote their health. In addition to game intensity, the game speed of current off-the-shelf exergames is still not suitable for senior adults. Therefore, intelligent recommended systems of game intensity, speed, or difficulty might be helpful for senior adults with various health conditions. Finally, senior adults also want to be fashionable and keep up with trends, not to be excluded by the market, so exergame developers may consider including senior adults’ physical and psychological needs to create age-friendly exergames that are more accessible. Future research could focus on investigating age-friendly game tutorials, developing approachable adventure games, creating adjustable game intensity levels, and designing game artwork for senior adults to enhance their exercise for better health.

## Abbreviations

WHO: World Health Organization

## Data Availability

All data generated and analyzed during this study are included in this published paper.
